# Accelerated Wound Healing in Minipigs by On-Site Production and Delivery of CXCL12 by Transformed Lactic Acid Bacteria

**DOI:** 10.3390/pharmaceutics14020229

**Published:** 2022-01-19

**Authors:** Emelie Öhnstedt, Hava Lofton Tomenius, Peter Frank, Stefan Roos, Evelina Vågesjö, Mia Phillipson

**Affiliations:** 1 Department of Medical Cell Biology, Uppsala University, 751 23 Uppsala, Sweden; emelie.ohnstedt@mcb.uu.se (E.Ö.); hava.lofton-tomenius@mcb.uu.se (H.L.T.); evelina.vagesjo@ilyapharma.se (E.V.); 2 Ilya Pharma AB, Dag Hammarskjölds Väg, 752 37 Uppsala, Sweden; peter.frank@ilyapharma.se; 3 Department of Molecular Sciences, Swedish University of Agricultural Sciences, 756 51 Uppsala, Sweden; Stefan.Roos@slu.se; 4 The Science for Life Laboratory, Uppsala University, 752 37 Uppsala, Sweden

**Keywords:** topical, *Limosilactobacillus reuteri*, wound measurement, active wound care, chemokines, three-dimensional imaging

## Abstract

Non-healing wounds are a growing medical problem and result in considerable suffering. The lack of pharmaceutical treatment options reflects the multistep wound healing process, and the complexity of both translation and assessment of treatment efficacy. We previously demonstrated accelerated healing of full-thickness wounds in mice following topical application of the probiotic bacteria *Limosilactobacillus reuteri* R2LC transformed to express CXCL12. In this study, safety and biological effects of a freeze-dried formulation of CXCL12-producing *L. reuteri* (ILP100) were investigated in induced full-thickness wounds in minipigs, and different wound healing evaluation methods (macroscopic, planimetry, 2D-photographs, 3D-scanning, ultrasound) were compared. We found that treatment with ILP100 was safe and accelerated healing, as granulation tissue filled wound cavities 1 day faster in treated compared to untreated/placebo-treated wounds. Furthermore, evaluation using planimetry resulted in 1.5 days faster healing than using 2D photographs of the same wounds, whereas the areas measured using 2D photographs were smaller compared to those obtained from 3D scans accounting for surface curvatures, whereas ultrasound imaging enabled detailed detection of thin epithelial layers. In conclusion, topical administration of the drug candidate ILP100 warrants further clinical development as it was proven to be safe and to accelerate healing using different evaluation methods in minipigs.

## 1. Introduction

The skin serves as an important barrier to the environment, and wounding of the skin rapidly initiates a healing process. How quickly the wound closes depends on wound size and location, and is also influenced by age, underlying medical conditions, nutritional status, and medications [1,2,3]. Occasionally, the healing process is impaired and the wound is referred to as non-healing if it has failed to heal within 3 months. Non-healing wounds are a growing medical problem associated with aging populations and the prevalence of metabolic diseases [2]. In addition to causing discomfort and pain, such wounds increase the risk of amputation due to infections and result in associated care costs that can account for over 3% of the healthcare budget in industrialized countries [4,5]. There are currently very limited options for active treatment, i.e., treatments that accelerate wound healing. Instead, available therapies aim to protect the wound and create a moist wound environment favorable for healing [6]. Effective drugs that actively promote healing of these complicated wounds would not only alleviate suffering and conserve healthcare resources, but also reduce the use of antibiotics. 

The complex process of wound healing can be divided into four consecutive and overlapping phases: hemostasis, inflammation, proliferation, and remodeling. During hemostasis, the bleeding is restricted by vasoconstriction followed by clot formation. Immune cells accumulate at the wound site during the inflammation phase and are important for fighting pathogens. It has become increasingly evident that the immune cells at the wound site also play a crucial part in orchestrating wound healing during the proliferative and remodeling phases during which granulation occurs, the wound is re-epithelialized, and the scar is formed. 

The development of treatments to accelerate wound healing is associated with many challenges, which explains the limited range of available options. For instance, topical administration of drug candidates is limited by the proteolytic microenvironment of the wounds, which greatly reduces bioavailability [7]. We recently developed a means to circumvent this issue by transforming a strain of the probiotic bacteria *Limosilactobacillus reuteri* R2LC (*L. reuteri* R2LC, previously known as *Lactobacillus reuteri* R2LC) to express murine or human CXCL12, which allows continuous expression of the protein at the wound site while inhibiting degradation of the chemokine [8]. Topical application of this genetically engineered *L. reuteri* R2LC was demonstrated to accelerate healing of full-thickness wounds in otherwise healthy or diabetic mice, and mice with peripheral hind limb ischemia, as well as to improve re-epithelialization using an ex vivo model of human skin disks [8]. This effect was proven to be macrophage-dependent, and both macrophage numbers and their transforming growth factor β (TGF-β) production increased by CXCL12-producing *L. reuteri* R2LC treatment, which ultimately resulted in increased proliferation of keratinocytes and accelerated wound healing [8]. 

Another challenge for drug development to increase wound healing is the translation from the most readily available animal models in rodents to the clinic. For instance, the healing of mouse skin wounds differs in many aspects from that in humans. The skin of rodents is loosely attached to the subcutaneous connective tissue and contains a thin muscle layer (panniculus carnosus), which enables the wound to contract by bringing the wound edges close together [9,10,11]. In contrast, wound closure in pigs and humans solely depends on the formation of granulation tissue and re-epithelialization, as wound contraction does not occur since the skin is firmly attached to the underlying connective tissue and lacks the required muscle layer [9,10,11]. In addition, non-healing wounds form a heterogenous group as they are the result of several underlying and complex conditions, making them impossible to fully replicate preclinically. The available non-healing wound models usually consist of wounds induced in an animal model of a primary condition associated with non-healing wounds, such as ischemia and diabetes. However, non-healing wounds have a more multifaceted pathophysiology and also depend on factors such as age and bacterial load [2,6,12]. 

Translation of preclinical protocols is moreover hampered by the current primary endpoints of clinical trials, as these do not reflect the wound healing process. The approved primary endpoints for clinical trials for chronic cutaneous ulcers and burn wounds, according to the FDA guidelines from 2006, are complete healing, facilitation of surgical closing, or reduced scarring [13,14]. The narrowness of these endpoints has been questioned by both investigators and clinicians, as they do not report the progression or quality of the wound healing process. Furthermore, additional information collected during monitoring of the healing process forms an untapped resource, which complements the conventional documentation of wound appearance and area [3]. An ongoing collaborative effort with the FDA therefore aims to identify new primary endpoints with improved clinical relevance and patient value [15], and has so far recognized reduced pain, fewer infections, and increased physical function/ambulation as the three most important parameters [16]. Based on these results and the work of the Wound Healing Endpoints Committee led by Driver et al., the FDA recently acknowledged five new endpoints [14]. 

The main objective of this paper was to increase the quality of wound healing evaluation by exploring and comparing classic as well as novel methods for the assessment of healing of induced, full-thickness wounds in minipigs. In parallel, the effect of the freeze-dried formulation of human CXCL12-producing *L. reuteri* R2LC, ILP100 a new-in-class drug candidate, on wound healing was evaluated in two separate cohorts of minipigs.

## 2. Materials and Methods

### 2.1. Study Design

Two separate studies were performed as part of the GLP toxicity program for ILP100; herein referred to as Cohort A and B, conducted two years apart. The primary objective of both studies was to assess safety and toxicity for regulatory compliance. The two studies also contained a number of complimentary technical and analytical exploratory endpoints which are reported herein. 

### 2.2. Animals

Cohort A was performed in 18 male, and Cohort B in 15 female Göttingen SPF minipigs (Ellegaard Göttingen Minipigs A/S, Dalmose, Denmark) at CitoxLabs (Ejby, Denmark). At cohort inclusion, the pigs were randomized to the different treatment groups, weighed 19–25 kg, and were between 7 to 11 months old. All experiments were approved by The Danish Veterinary and Food administration Council (Ethical permit number; 2015-15-0201-00713).

### 2.3. Limosilactobacillus reuteri R2LC Encoding Human CXCL12

A strain of probiotic bacteria *Limosilactobacillus reuteri* R2LC (*L. reuteri* R2LC) genetically engineered to encode human CXCL12 1 alpha has been designed as reported elsewhere [8], and developed in a freeze-dried formulation as the drug candidate ILP100. In brief, the sequences encoding the human chemokine CXCL12 1 alpha were inserted into an expression vector, after which the constructs were transformed into *L. reuteri* R2LC. The CXCL12 expression is induced by the addition of an inducing peptide, SppIP, resulting in the transformed *L. reuteri* R2LC expressing human CXCL12 following activation (for details [8]). 

### 2.4. Wound Induction

The animals were anesthetized on day 1. The anesthesia protocols differed slightly between Cohort A and Cohort B: both cohorts received, an intramuscular injection a mixture of Zoletil 50^®^Vet., (125 mg tiletamine and 125 mg zolazepam), 20 mg xylazine/mL (6.25 mL), 100 mg ketamine/mL (1.25 mL) and 10 mg butorphanol/mL (2.5 mL). In addition to these injections, the minipigs in Cohort A received an intramuscular injection of 40 mg azaperone (1 mL/20 kg) and 1 mg atropine/mL (0.05 mL/kg). The minipigs in Cohort B did not receive the azaperone/atropine injection and the anesthesia was instead maintained by isoflurane. 

Prior to wound induction, the back of the animal was shaved, washed, and disinfected. Two to three circular full-thickness wounds (20 mm diameter, area 3.14 cm^2^) were induced on each side of the spine on the back of each animal. 

For prevention of post-surgical pain due to wound induction, the animals were treated with fentanyl dermal patches (Cohort A 75 µg/h, Cohort B 50 µg/h) and a daily intramuscular injection meloxicam (Cohort A 20 mg/mL, 0.02 mL/kg, Cohort B 5 mg/mL, 0.08 mL/kg) for up to 72 h. 

### 2.5. Blood Sampling

In Cohort A, blood samples were drawn from the jugular vein before the start of treatment, on day 4 and on the day of the euthanization. In Cohort B, the blood samples were drawn from the jugular vein or the ear vein catheter before the start of treatment, on day 3 and on the day of the euthanization. All animals were fasted overnight with free access to water before blood sampling. For both cohorts, conventional panels for hematology and clinical chemistry were assessed.

### 2.6. Urinalysis

Before the start of treatment and before the termination of treatment, urine was collected from all animals. Urine samples were collected overnight from the stainless-steel tray under the stall and were assessed on conventional panels for urinalysis.

### 2.7. Histopathology

At euthanization, necropsy samples from all animal tissues were taken for histological processing (Table A1). The specimens were embedded in paraffin and cut at a nominal thickness of approximately 5 µm, stained with haematoxylin and eosin, and examined under a light microscope. Histological alterations were graded on a 5-level scale (minimal, mild, moderate, marked, and severe) by a pathologist, and selected slides were examined by a peer-reviewing pathologist.

### 2.8. Wound Treatment 

**Figure 1:**
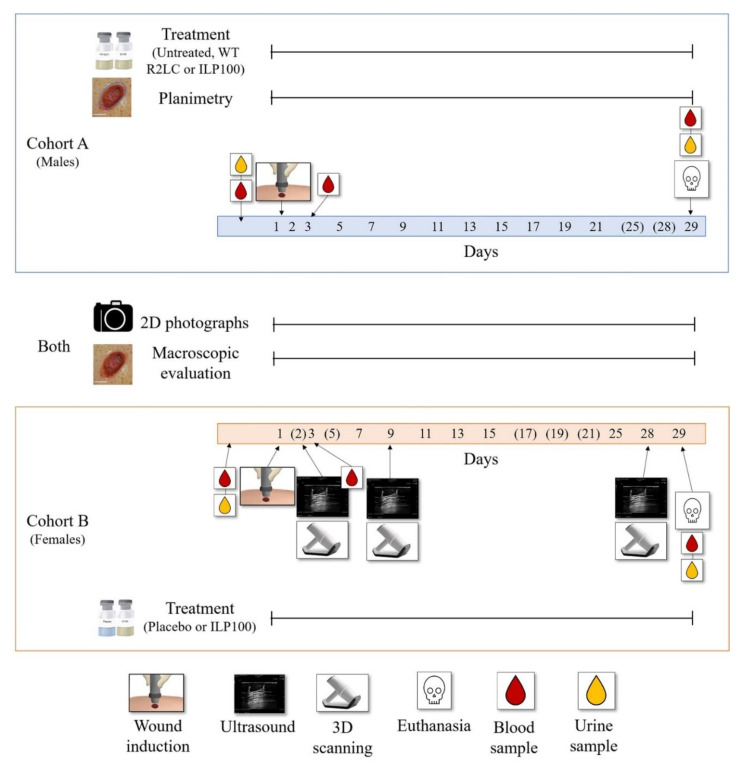
Schematic illustration of the protocol design. The study consists of two cohorts: one in males (Cohort A, blue box) and one in females (Cohort B, orange box). In the illustration, the numbers in the filled blue (Cohort A) and orange (Cohort B) boxes indicate the days where 2D photographs, macroscopic evaluation, treatment, and planimetry (only Cohort A) were performed. No 2D photographs, macroscopic evaluation, or planimetry were performed on the days in parenthesis. Time points for wound induction, collection of blood (for hematology, clinical chemistry, plasma levels of CXCL12 and SppIP, and for CFU counts of ILP100), collection of urine samples, and for additional imaging (only Cohort B) with ultrasound and 3D scanning are indicated with arrowed symbols.

The wounds were treated and the dressings were changed on days 1, 2, 3, 5, 7, 9, 11, 13, 15, 17, 19, 21, 25, and 28 following wound induction (Figure 1). Cohort A comprised three subsets in which the wounds: (i) did not receive any treatment, (ii) were treated with wild type *L. reuteri* R2LC (500 µL, 2.5 × 10^9^ CFU/wound), (iii) were treated with ILP100 (100 µL, 7 × 10^9^ CFU/wound). In Cohort B, the wounds were treated either with (i) placebo (500 µL) or (ii) ILP100 (500 µL, 2.5 × 10^9^ CFU/wound). Before treatment of the wounds, the freeze-dried formulations were reconstituted in buffer and activated with abundant amounts of SppIP (100 to 1000 ng/mL). 

### 2.9. Wound Evaluation

The wounds were macroscopically evaluated and photographed in a standardized manner at day 1 (only photo), 2, 3, 5, 7, 9, 11, 13, 15, 17, 19, 21, and 28 in Cohort A, and at day 1 (only photo), 3, 7, 9, 11, 13, 15, 21, 25 and 28 in Cohort B (Figure 1). The macroscopic evaluation performed on-site included scoring of granulation, presence of hypergranulation, wound edge inflammation, surrounding skin inflammation, hemorrhaging, and exudation. The scoring ranged from 0—not present, 1—minimal, 2—slight, 3—moderate, and 4—marked. 

#### 2.9.1. Two-Dimensional Photographs of Wounds

Two-dimensional (2D) photographs were taken in a standardized manner with a flash using the same camera and at a fixed distance with a 5.5 cm × 5.5 cm frame placed around the wound. From the photographs, area measurements were performed using ImageJ2 software (National Institutes of Health, Bethesda, MD, USA) where the frame in the photos served as the scale.

#### 2.9.2. Planimetric Assessments of Wounds

In Cohort A, the wounds were measured using planimetry days 2, 3, 5, 7, 9, 11, 13, 15, 17, 19, 21, and 28. For the planimetry assessment, a sterile transparent sheet was placed on top of the wound, on which both the area of the wound and the newly formed epithelia were outlined. In the wound, the area covered with granulation tissue was outlined and the remaining wound area was marked as unspecific tissue. The sheets were later analyzed using PictZar Pro (7.5.1) (Advanced Planimetric Services, Elmwood Park, NJ, USA).

#### 2.9.3. Three-Dimensional Scanning of Wounds

In Cohort B, three-dimensional wound measurements were carried out using a stereoscopic optical system, a Cherry Imaging platform consisting of a hand-held 3D scanner, and TraceTM version 5 software (Cherry Imaging, Yokneam, Israel, 2019). The 3D scanner acquires thousands of images with a speed of 15 frames/second at a resolution of 100 µm, that are rendered into a 3D surface [17]. For each pig, three of the wounds were scanned on days 2, 9, and 28. The wound margins were manually marked on the 3D surfaces created in the TraceTM software, and the program then calculated the area, volume, and depth of the wound. Wound depth was designated as the average of 10% of the measured spots with the deepest values. 

#### 2.9.4. Ultrasound Imaging of Wounds

In Cohort B, the three wounds that were 3D scanned were imaged using ultrasound on days 2, 9, and 28 following wound induction using Arietta V60 with linear probes L64, 5–18 MHz. The probe was placed in the same direction on all occasions for all wounds, in order to produce scans that visualized one transversal section of the entire wound. Scanning directly on the wound was only possible using saline flushed into the wound cavity (Days 2 and 9). On Day 28, the wounds were completely healed and epithelialized, and ultrasound gel was used instead of saline. 

### 2.10. Data Management and Statistics

Some data points from 2D photographs (123 of 2058) and 3D scans (32 of 135) are missing due to poor image quality, precluding reliable analysis. In addition, ultrasound images from eleven minipigs on day 2 were lost due to software failure during data transfer. All four wounds from one minipig from Cohort A in the ILP100 treated group were excluded since they all became infected. All data are presented as mean ± SEM and/or with individual dots for each wound. Each wound was treated as an independent variable in the statistical analysis. Comparisons between area measurements achieved with different methods on the same wounds were performed with a Wilcoxon signed rank test. For comparison with unpaired values, the Mann-Whitney or Kruskal-Wallis test with Dunn’s multiple comparison test was used. Jonckheere’s trend test was used to assess if there were a trend between ordinal independent variables and continuous variables. *p* < 0.05 was considered significant. All statistical analyses were performed using GraphPad Prism 9.0.1. (GraphPad Software, San Diego, CA, USA, 2021) 

## 3. Results

### 3.1. Evaluation of Methods Assessing Wound Granulation, Re-Epithelialization and Area

Wound healing was assessed in minipigs using consecutive measurements of areas and volumes of induced wounds, as well as of the formed scars. Areas of wounds and early scars were measured and analyzed by three approaches: 2D photographs with ImageJ2 software, planimetry with PictZar Pro software, and 3D scans with the TraceTM software (Figure 1). Wound diameters were also measured by 2D photographs, 3D scans, and ultrasounds. Wound and scar volumes were assessed by 3D scanning, and all wounds were evaluated macroscopically for assessment of granulation tissue at different time points following induction.

**Figure 2:**
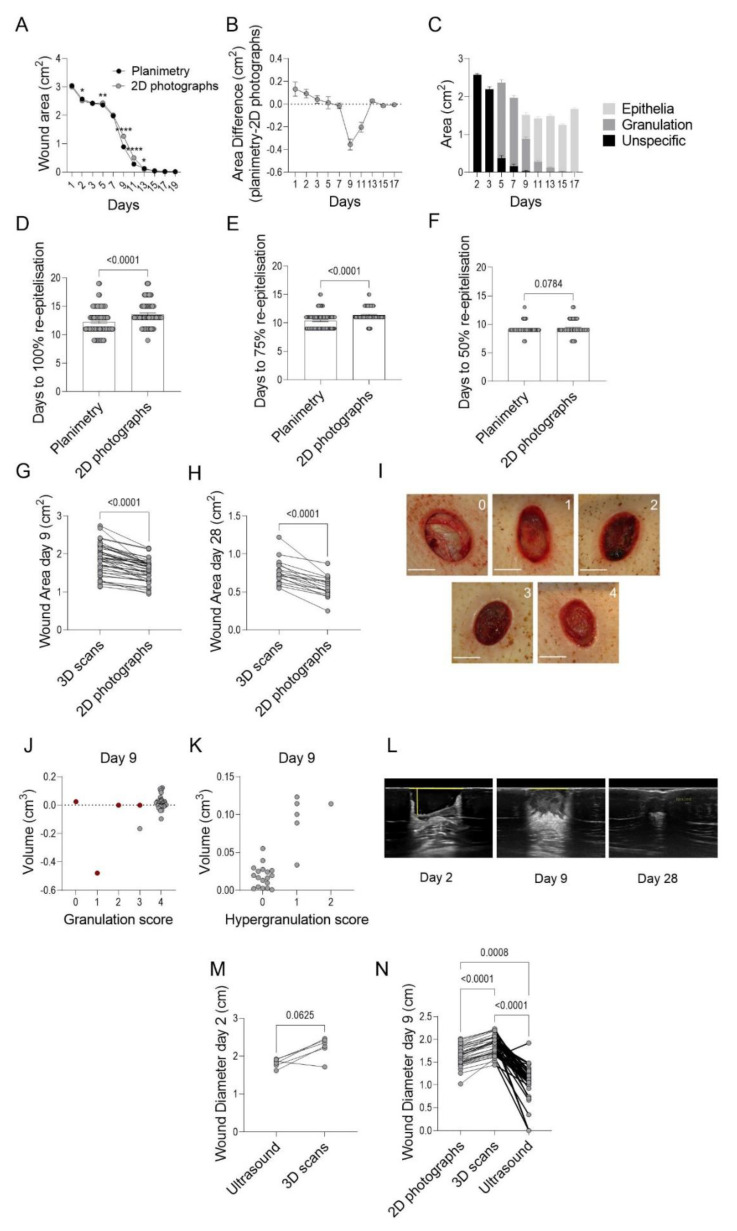
Comparisons of different wound assessment methods. Wound areas from Cohort A were measured by on-site planimetry (**A**) and from 2D photographs (**B**), *n* = 18, *N* = 93–96), and the areas were compared to reveal method-dependent differences. From the planimetric data, the areas of newly formed epithelia, granulation tissue, and unspecific tissue for all wounds in Cohort A (**C**) were retrieved (*n* = 18, *N* = 96). Furthermore, days to 100% (*n* = 18, *N* = 96), 75% (*n* = 18, *N* = 91), and 50% (*n* = 18, *N* = 91) re-epithelialisation of all wounds were assessed with planimetry and from 2D photographs (**D**–**F**) in Cohort A. In Cohort B, method-dependent differences between area measured with 3D scanning and from 2D photographs on day 9 (**G**), *n* = 17, *N* = 42), and day 28 (**H**), *n* = 6, *N* = 17) were assessed. In addition, the formation of granulation tissue was assessed macroscopically by scoring from 0 to 4 (scale bar 1 cm) (**I**). To assess correlations between macroscopic evaluations and volume measurements from the 3D scans at day 9, granulation score (**J**) and hypergranulation score (**K**) was plotted against volumes generated from the 3D scans of the respective wound (*n* = 17, *N* = 42), where red dots indicate clot formation in the wound. On days 2, 9, and 28, the wounds in Cohort B were imaged with ultrasound and representative images are shown (**L**), where the yellow lines delineate measured diameters and depth showing that the wounds are fully epithelialized and considered healed at d28. Method-dependent differences were assessed by comparing the diameter of each wound measured from images acquired by ultrasound or 3D scanning day 2 (**M**) and by ultra-ound, 3D scanning, or from 2D photographs on day 9 (**N**). Statistic comparisons were made using Wilcoxon signed rank test (**A**,**D**–**F**,**H**,**I**,**M**), Jonckheere’s trend test (**J**) and Friedmans test with Dunn’s multiple comparison test (**N**) (* *p* < 0.05, ** *p* < 0.01, **** *p* < 0.0001).

In Cohort A, wound areas were assessed using planimetry and 2D photographs according to standardized protocols. On days 2, 5, 9, 11, and 13 following wound induction, there were statistically significant differences between the measured mean wound areas (93–96 wounds per time point) using planimetry or 2D photographs. However, of these time points, only days 9 and 11 had area differences exceeding 0.1 cm^2^, and the planimetry-measured areas were 0.36 (± 0.05) cm^2^ and 0.20 (± 0.04) cm^2^ smaller for the respective days compared to the areas measured from 2D photographs (Figure 2A,B). Epithelialization was first noted on day 9 (Figure 2C) and might account for the observed area differences, as it is more difficult to detect thin epithelial layers from the 2D photographs acquired with a flash compared to those assessed by planimetry. In fact, the time to 50%, 75%, and 100% re-epithelialization differed depending on the method used, as the time to 100% re-epithelialization occurred on average 1.5 days later (*p* ≤ 0.0001) when assessed by 2D photographs (planimetry: 12.2 ± 2.3 days, 2D photographs 13.6 ± 2.1 days, Figure 2D). Similarly, the 75% and 50% re-epithelialization were reached 1.0 day (*p* ≤ 0.0001) and 0.2 day (*p* = 0.08) earlier, respectively, when assessed by planimetry compared to 2D photographs (Figure 2E,F). 

To allow measurements of the wound area, depth, and volume, as well as the height and volumes of scars, a technology with stereoscopic scanning generating 3D surface models was utilized in Cohort B. When the areas obtained from 3D scanning was compared to those obtained from the 2D photographs of the same wounds on day 9 and day 28, it became evident that smaller wound areas were detected by 2D photographs when compared to those from 3D scans (day 9: 16 ± 8% smaller, *p* ≤ 0.0001, day 28: 27 ± 12% smaller, *p* < 0.0001, Figure 2G,H). 

The formation of granulation tissue in the wound cavity is a prerequisite for re-epithelialization as it enables epithelial cell migration and wound closure (Figure 2I). The 3D scanning of wounds results in a negative volume corresponding to the wound cavity, while a positive volume depicts clot formation or hypergranulation, i.e., granulation tissue elevated beyond the level of the surrounding skin. The 3D scans were complemented with a macroscopic evaluation of the wounds before each treatment, and the wounds were scored (0–4) for the formation of granulation tissue, where higher scores indicate that a larger extent of the cavity is filled with granulation tissue. The granulation scoring and 3D volume measurements at day 9 were then plotted against each other to test for correlation. On day 9, the majority of the wounds had a granulation score of 4 and a wound volume close to 0 (Figure 2J). If wounds with blood clots were excluded (red dots in graphs), the wound with the largest cavity was the one receiving a lower granulation score, as expected. Due to the fast formation of granulation tissue in the induced wounds, 3D scanning should be performed at earlier time points to evaluate if those results correlate with the macroscopic observations. Interestingly, all wounds that were scored as hypergranulating from the macroscopic evaluation also showed a positive volume when measured by the 3D scanning, and a significant trend (*p* = 0.0001) towards higher hypergranulation scores with increasing volume was demonstrated using Jonckheere’s trend test (Figure 2K). 

Thus, wound healing can be evaluated using several parameters, and one method is not enough for assessing all aspects of the healing process. As demonstrated above, different methods might give different results when measuring the same parameters. Therefore, these differences should be taken into account when making informed decisions on which method to use in designing controlled studies assessing wound healing in animals and humans.

### 3.2. Treatment with CXCL12-Producing L. reuteri R2LC Accelerates Wound Healing

**Figure 3:**
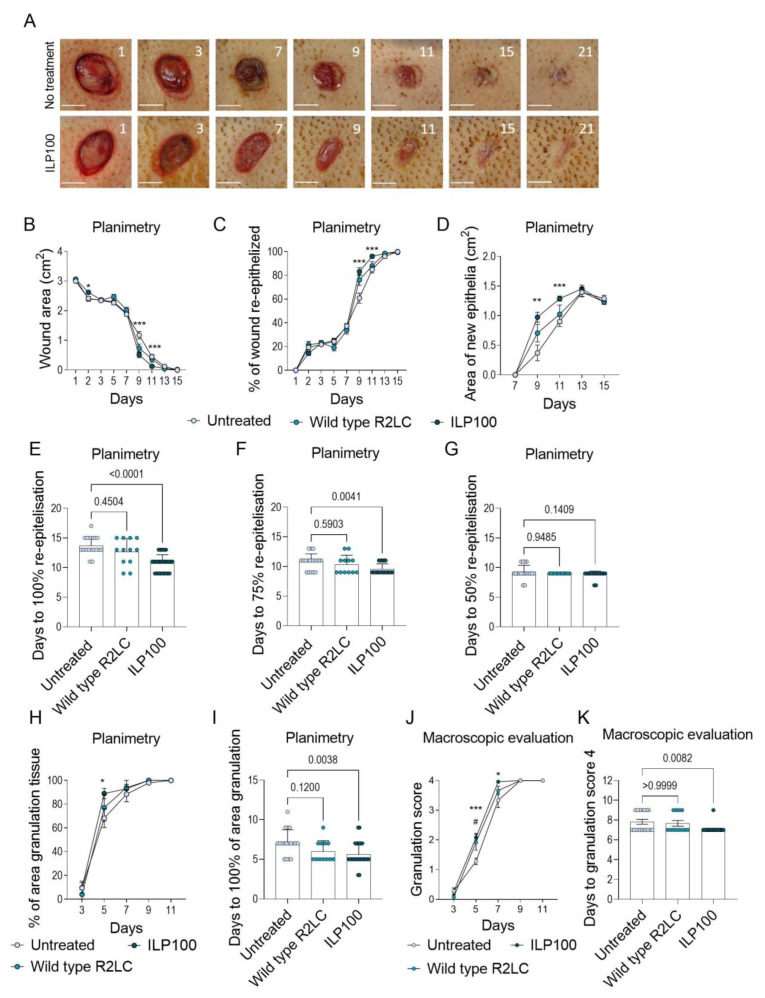
Wound healing assessed by planimetry and macroscopic evaluation in cohort A. (**A**) shows representative photographs of healing over time where the inserted numbers depict days post-induction and the scale bar corresponds to 1 cm. Wound healing was assessed by planimetry detecting wound area (**B**), % of the wound being re-epithelized (**C**), and area of newly formed epithelia over time (**D**). Days to 100% (**E**), 75% (**F**), and 50% (**G**) re-epithelization of the wounds were also assessed. In addition, granulation tissue formation was measured as wound area covered by granulation tissue (**H**) and days to 100% of the wound area being covered by granulation tissue (**I**) using planimetry, as well as macroscopically as granulation score (0–4) day 3 to 11 (**J**) and days to granulation score 4 (**K**). The animals and wounds were divided into the following groups: Untreated (*n* = 3, *N* = 18), Wild type R2LC (*n* = 3, *N* = 12), ILP100 (*n* = 5, *N* = 26). Statistic comparisons between groups were made using Kruskal-Wallis test with Dunn’s multiple comparison test (**B**–**K**). For (**B**–**D**,**H**,**J**) one test was performed for each time point. * Indicates the difference between untreated and ILP100 and # difference between untreated and wild type R2LC. (* *p* < 0.05, ** *p* < 0.01, *** *p* < 0.001).

The biological effects of freeze-dried human CXCL12-producing *L. reuteri* R2LC (ILP100) following topical treatment of wounds were then assessed and compared to untreated wounds or wounds receiving placebo or wild-type *L. reuteri* R2LC (Figure 3A). Directly prior to treatment of the wounds with a dose of 10^9^ colony forming units (CFU) per wound, the freeze-dried bacterial formulation was reconstituted, and expression was induced by the addition of abundant amounts of SppIP (100–1000 ng/mL supplied in the reconstitution buffer). 

**Figure A1:**
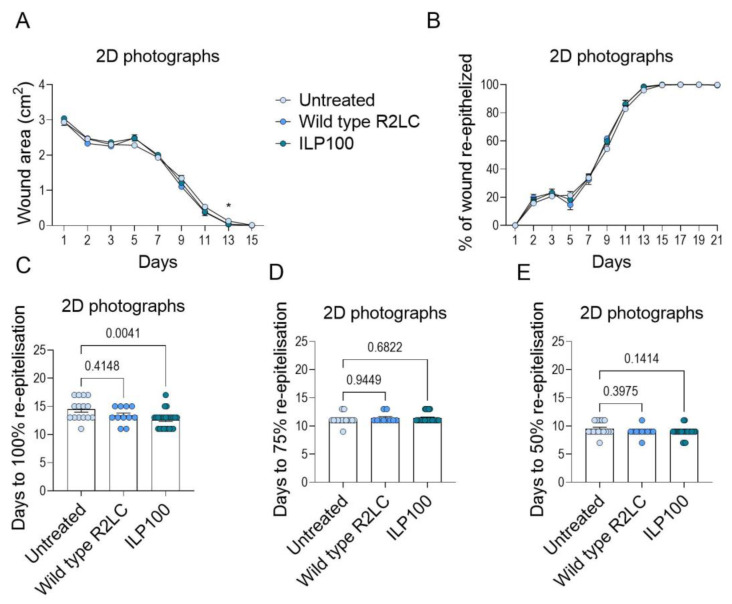
Wound healing assessed from 2D photographs for Cohort A. Wound area and wound healing over time were measured from 2D photographs in Cohort A (**A**,**B**), and days to 100% (**C**), 75% (**D**), and 50% (**E**) re-epithelization were assessed. (**A**,**B**): Untreated (*n* = 3, *N* = 18), Wild type R2LC (*n* = 3, *N* = 12), ILP100 (*n* = 5, *N* = 26). (**C**–**E**): Untreated (*n* = 3, *N* = 18), Wild type R2LC (*n* = 3, *N* = 11), ILP100 (*n* = 5, *N* = 25). Kruskal-Wallis test with Dunnett’s multiple comparison test (**A**–**E**). * *p* < 0.05.

The biological effect of ILP100-treatment was demonstrated in Cohort A by accelerated re-epithelialization and increased formation of granulation tissue as compared to untreated wounds (Planimetry). Accelerated re-epithelialization by ILP100 was observed as reduced wound area and increased percentage of the wounds being re-epithelialized when compared to untreated wounds and wounds treated with wild-type *L. reuteri* R2LC (Planimetry, Figure 3B,C). In addition, larger areas of newly formed epithelia were demonstrated following ILP100-treatment on days 9 and 11, and the treated wounds became fully re-epithelialized 3 days earlier than untreated wounds (Planimetry, Figure 3D,E), even though no differences were detected for the time leading to 50% or 75% re-epithelialization of the wound area (Planimetry, Figure 3F,G). The accelerated re-epithelialization demonstrated by planimetry was also supported by assessments from the 2D photographs, as the ILP100-treated wounds were fully epithelized almost 2 days faster than the untreated wounds (12.6 ± 0.4 days versus 14.4 ± 1.4 days, respectively, Figure A1). However, no differences were detected between treatments when absolute wound size, percent re-epithelialized wound area, or time to 50% or 75% re-epithelialization were analyzed in the 2D photographs. The discrepancy observed for results obtained by planimetry and 2D photographs were in line with our evaluation that planimetry reported faster wound healing compared to 2D photographs (Figure 2D). In addition, the formation of granulation tissue was accelerated by the ILP100 treatment at day 5, and the time to complete wound coverage of granulation tissue occurred 1.5 days earlier in the ILP100-treated wounds when compared to untreated wounds (Planimetry, Figure 3H,I). This observation was strengthened by the macroscopic evaluation, as ILP100-treated wounds received higher average scores for the assessment of granulation, starting at day 5 (Figure 3J,K). 

**Figure 4:**
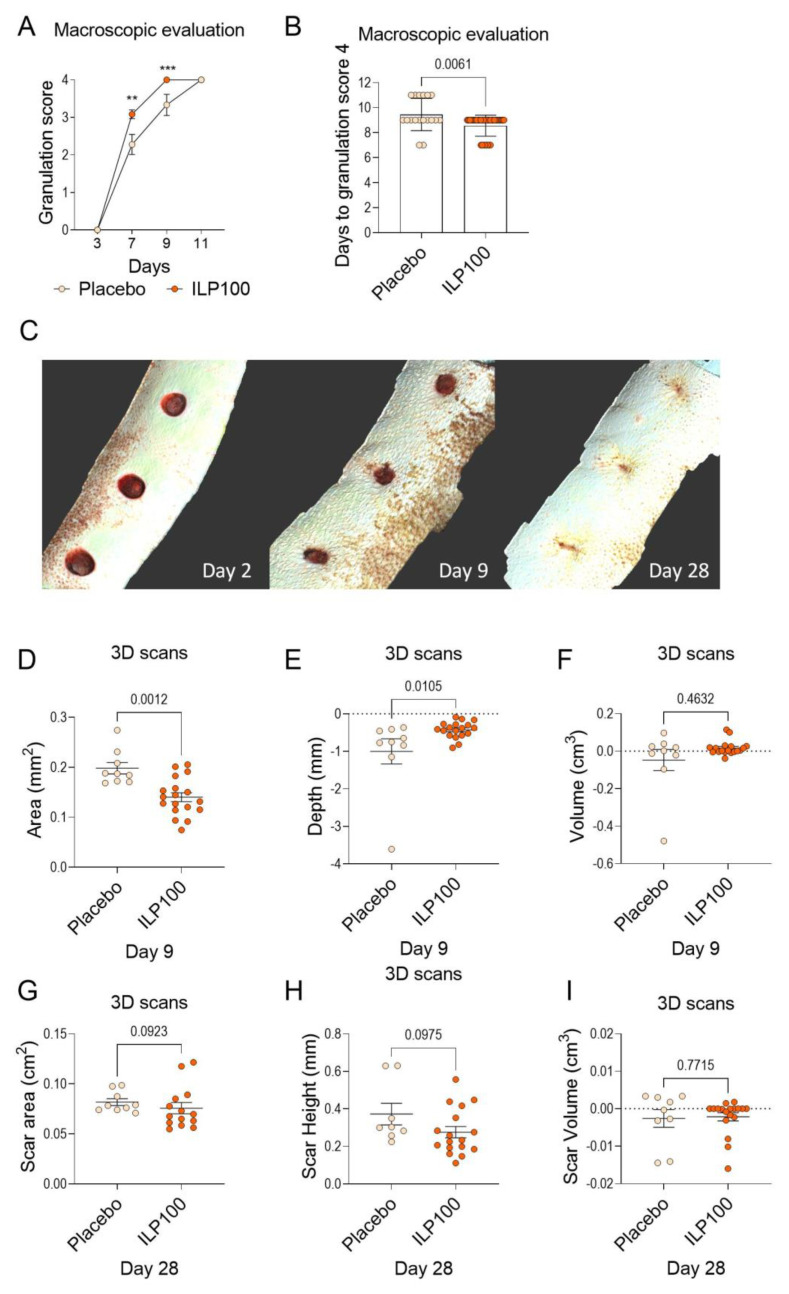
Wound healing assessed by macroscopic evaluation and from 3D scans in Cohort B. Wounds in Cohort B were macroscopically evaluated for granulation tissue formation by granulation score day 3 to 11 (**A**) and days to granulation score 4. ** *p* < 0.01, *** *p* < 0.001 (**B**) (Placebo: *n* = 3, *N* = 18, ILP100: *n* = 6, *N* = 36). The healing was also assessed using 3D scans, where panel (**C**) shows representative projections of 3D scans from day 3, day 9, and day 28. From the 3D scans, wound area, wound depth, and wound volume were measured (**D**–**F**) for placebo and ILP100-treated wounds day 9 (Placebo: *n* = 3, *N* = 9, ILP100: *n* = 6, *N* = 18). On day 28 when the wounds were healed, scar area, scar height, and scar volume (**G**–**I**) were measured from 3D scans, (Placebo: *n* = 3, *N* = 9, ILP100: *n* = 5–6, *N* = 14–18). Statistic comparison between the groups were made using Mann-Whitney tests (**A**,**B**,**D**–**I**).

**Figure A2:**
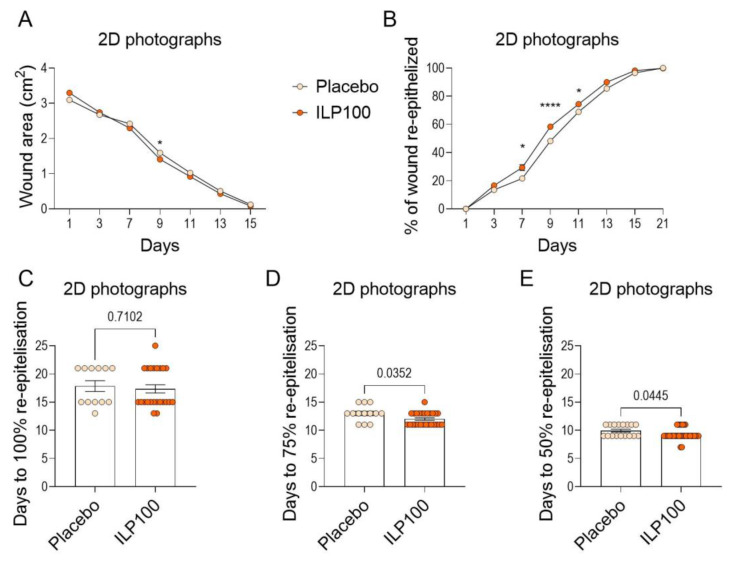
Wound healing assessed from 2D photographs for Cohort B. Wound area and wound healing over time measured from 2D photographs in Cohort B (**A,B**). From these measurements, days to 100% (**C**), 75% (**D**), and 50% (**E**) re-epithelization were assessed. (**A**,**B**); Placebo (*n* = 3, *N* = 18), ILP100 (*n* = 6, *N* = 36). (**C**–**E**): Placebo (*n* = 3, *N* = 12–17), ILP100 (*n* = 6, *N* = 23–35). Mann-Whitney (**A**–**E**). * *p* < 0.05, **** *p* < 0.0001.

In Cohort B, macroscopic evaluation again demonstrated increased granulation of ILP100-treated wounds at day 7 and day 9 when compared to placebo (Figure 4A,B), whereas no granulation scoring was performed at day 5 as part of the protocol. Planimetry was not performed in Cohort B, but data from 2D photographs revealed an increased portion of re-epithelialization of wounds following ILP100-treatment at day 7 and day 9, resulting in the ILP100-treated wounds reaching 75% and 50% re-epithelialization area faster than the placebo-treated wounds (Figure A2B,D,E). No differences in wound area or time to complete re-epithelialization between treatments could, however, be detected in analyses from the 2D photographs (Figure A2A,C). 

The wounds in Cohort B were also imaged by the 3D scanner on three occasions (day 2, 9, and 28, Figure 4C). In accordance with the observations from the 2D photographs, a reduced wound area was observed on day 9 for the ILP100-treated wounds using the 3D scans (Figure 4D). The 3D scans also revealed a reduced depth (calculated as the mean of the deepest 10% of measurements of the wound) in the ILP100-treated wounds compared to placebo (Figure 4E), even though no statistical differences in wound volumes were observed (Figure 4F). Early scarring was evaluated using the 3D scans at day 28, where no statistical differences could be observed between treatments, even though a trend of reduced scar area (*p* = 0.0923) and reduced scar height (*p* = 0.0975, Figure 4G–I) were detected for the ILP100 treated wounds. 

Taken together, topical ILP100 treatment to induced full-thickness wounds accelerated wound healing in both male and female minipigs.

### 3.3. Safety Assessments

Safety was assessed for all treatments, and there were no reported observations that could be linked to treatment in conventional hematology-, clinical chemistry-, or urine analysis in any sample. In addition, no histopathological findings (for examined samples see Table A1) were reported by the respective pathologist, and the healing of all wounds was categorized as advanced. 

Local tolerability was assessed by scoring inflammation of wound edges and surrounding skin. Overall, there were no differences detected between the treatments over time, though a slight increase in inflammation of the surrounding skin was observed in ILP100 treated wounds at day 29 in Cohort A, with an increase from a 0.0 (± 0.0) score in untreated wounds to 0.6 (± 1.0) (Table A2).

Small differences were observed in the hemorrhage scoring. In Cohort A, hemorrhage scoring was slightly increased for wild type- and ILP100-treated wounds compared to untreated wounds at day 7 (untreated: 0.3 ± 0.6, representing non- to minimal bleeding, wild type R2LC; 1.4 ± 0.5, and ILP100: 1.2 ± 0.6, representing minimal to slight bleeding, Table A3). Increased bleeding was also observed on day 7 in Cohort B, with an average hemorrhage score of 0.4 ± 0.5 for placebo-, and 0.9 ± 0.5 for ILP100-treated wounds. In contrast, decreased bleeding was observed for treated wounds at day 9 in Cohort A and at day 3 and 11 in Cohort B (Table A3). In Cohort A, less exudate was observed on days 2–5 in wild type R2LC- and ILP100-treated wounds compared to untreated wounds (Table A4), but this was not observed in Cohort B. Furthermore, no elevated hypergranulation scoring was observed for the ILP100-treated wounds in either cohort, while wild type R2LC-treated wounds received a slightly increased score compared to untreated wounds in Cohort A (Table A5). 

Thus, the safety assessment concluded that topical treatment with ILP100 to induced full-thickness wounds in minipigs was both safe and well-tolerated. 

## 4. Discussion

Despite being a significant societal burden in industrialized countries, available treatment options for non-healing wounds are today very limited. This study investigates the biological effects of the drug candidate ILP100 on induced wounds in minipigs by evaluating different and novel methods for the assessment of wound healing. Of the methods evaluated, we found that planimetry reported reduced wound areas (day 9 and 11) and faster healing when compared to 2D photographs, which in turn reported smaller wound areas than 3D scans. Wounds treated with ILP100 demonstrated accelerated healing by advanced re-epithelialization, as revealed by planimetry, 2D photographs, and 3D scans, in addition to higher granulation scores and increased area of granulation, as measured by planimetry. 

For successful translation of preclinical projects, the clinical relevance of the models used is essential. The most widely used experimental animals are inbred mice due to their small size, as well as the wide palette of available genetically modified strains. However, rodent skin contains a muscle layer (panniculus carnosus) that enables the contraction of wounds, which does not exist in human skin and complicates translation. In contrast, pig skin not only lacks the contractile muscle layer but also resembles human skin with its sparse haircoat, firm attachment to underlying connective tissue, and epidermal turnover time [11,18,19]. When the translational success was evaluated in 25 wound healing studies, the agreement between the pre-clinical and clinical outcome was higher for pre-clinical evaluation in pigs (78%) than in smaller mammals (53%) or using in vitro studies (57%) [20]. In the current study, our previous observation of accelerated wound healing in mice treated with CXCL12-producing *L. reuteri* R2LC was confirmed to also occur in minipigs, even though with different kinetics. 

In addition to inter-species differences in skin anatomy, the complexity of the wound healing process, and particularly that of non-healing wounds and associated underlying pathologies, adds another challenge to the development of new efficient therapies. Indeed, during the last decades, many treatments that passed the safety requirements of Phase I clinical trials later fail to show consistent efficacy in wound healing in Phase II or III [21]. This is likely to depend on several factors, such as differences in the wound healing process between wound types, as well as difficulties in ensuring bioavailability following topical administration due to the high levels of degrading enzymes in the wound environment. Another factor for the limited success of clinical trials is that the only accepted primary endpoint to date is complete wound healing, reported as the time to heal, or the fraction of healed wounds at a relevant time point [16]. However, healing of wounds not only involves a reduction of the wound area through re-epithelialization, but also requires regeneration of tissue in the wound cavity, namely the formation of granulation tissue. In fact, the absence of healthy granulation tissue is a characteristic of non-healing wounds [3,22]. For this reason, solely evaluating wound healing by repeated measurements of wound area does not readily account for the wound healing process. In addition, the depth of the wound has been shown to be a predictor of its healing rate [23], as well as being associated with the risk of amputation in diabetic foot ulcers [24,25]. Therefore, assessment of the granulation tissue and depth measurements are part of many of the assessment tools that have been developed for non-healing wounds, such as Pressure Ulcer Scale for Healing (PUSH), Sussman Wound Healing Tool (SWHT), and Bates-Jensen wound assessment tool (BWAT) [23,26,27,28,29]. Of these tools, only BWAT considers the amount of granulation tissue while the others only assess the presence or absence of healthy granulation tissue. Other factors such as exudation, inflammation, and the presence of necrotic tissue or slough may also give an indication on how the healing is progressing [30,31,32]. Thus, extensive efforts have been made to identify appropriate new primary endpoints for wound healing studies [15,16,33]. Even though the primary endpoint of complete healing remains, the FDA recently announced that it is open to discussing new primary endpoints, including (1) Percentage area reduction (PAR), (2) Reduced infection, (3) Reduced pain/reduced analgesia usage, (4) Increased physical function and ambulation, and (5) Quality of life [14].

Translation of preclinical wound therapies is also limited by the fact that there is no current gold standard for evaluating wound healing. For clinical practice, several different wound healing assessment scales have been developed, of which the two more commonly used are the Pressure Ulcer Scale for Healing (PUSH) and Bates-Jensen wound assessment tool (BWAT) [23,26,27,28]. As mentioned above, these scales score the wounds based on several parameters, including area, depth, and tissue type. However, the dimensions used comprise linear measurements, where the surface area is calculated by measuring the length and the width of the wound (area = length × width), while wound depth is based on one or a few measurements by a ruler. While these linear measurements are easy to execute, the calculated areas have been shown to overestimate the wound area by up to 40% [34]. More precise measurements can be achieved by measuring the area within the wound boundaries, as in this study for planimetry, 2D photographs, and 3D scans. Planimetry and wound area measurements from 2D photographs have been demonstrated to have good inter- and intra-investigator reliability [34,35,36]. However, while planimetry and 2D photographs reported similar wound areas for most time points in the current study, smaller wounds were detected on days 1 and 2 using 2D photographs compared to planimetry, while the opposite was found on days 9 and 11. The difference observed days 1–2 is in agreement with a previous study comparing the same type of methods where mean wound areas directly after induction (0.10–2.20 cm^2^) were smaller when measured using 2D photographs compared to planimetry [35]. The differences in measured wound area detected on days 9 and 11 coincide with when newly formed epithelia can first be detected using planimetry. This observation demonstrates an inherent problem with wound healing assessments, namely the definition of the wound boundary. If the wound boundary is delineated by the newly formed epithelia, only methods enabling epithelial detection will identify this new edge, and thereby report smaller wound areas. While the newly formed epithelia are easily identified using conventional histopathology that does not allow studies over time, it requires high-resolution images for repeated evaluations. The limited resolution of the 2D photographs is probably causing the observed discrepancies in wound areas when assessed by planimetry or 2D photography at the time of formation of thin epithelial layers (day 9–11), even though the individual definition of wound boundaries by the evaluators cannot be excluded. Poor resolution of 2D photographs was a limiting factor when wound diameters obtained from 2D photographs or ultrasound images were compared, as the latter technique resulted in significantly shorter diameters. 

However, when designing the protocol of a clinical trial assessing wound healing, it is not only the accuracy of the method, but also several other factors relevant to wound healing assessment, that need to be considered. For instance, 2D photographs offer traceability and a reduced risk of infection, as contact with the wounded area is not required. In addition, neither planimetry nor 2D photographs generate wound depth or volume, which traditionally have required more invasive methods, including a cotton-tipped applicator for the former or filling the wound cavity with saline or alginate gel for the latter [23]. To circumvent the risk of causing infections, 3D non-invasive wound measuring techniques have been developed, but have been limited by costly equipment requiring special training [36]. In recent years, more user-friendly systems have been launched, some of which have been shown to produce reliable area measurements [37,38,39], while depth and volume measurements are underestimated in some evaluations [37,38]. Here, the Cherry Imaging system comprising a stereoscopic 3D scanner and associated TraceTM software was assessed for healing of full-thickness wounds in minipigs. This system has not previously been used for wounds but has been evaluated in scar models and in patients with hypertrophic and atrophic scars [40], where it demonstrated good intra- and inter-observer repeatability with no difference detected for depressed or elevated scars [40]. When applying this system on induced wounds, we found that 3D scans resulted in larger areas when compared to areas obtained from 2D photographs. This difference could be due to the fact that only the 3D scans take the curvature of the pigs’ backs into consideration. A study comparing the areas of human wounds with various sizes (0.3–36.1 cm^2^) and etiology measured either by 3D images captured with another stereoscopic imaging device or 2D photographs analyzed using ImageJ, did not find any differences between methods [39]. The large range of wound size and the heterogeneity of the assessed wounds might also have contributed to a difference not being detected in this study. In addition, the same study reports that volume measurements acquired from the 3D images correlated well with volume measured by gel application [39].

Immune cells are known to contribute to the distinct phases of the wound healing cascade by different means [41,42]. While innate bactericidal functions are crucial in the early stages following wounding, the tissue restorative functions of predominantly macrophages drive the healing and remodelling processes during the later phases. The healing process is orchestrated by a cascade of growth factors, chemokines, and cytokines, and delivery of these to the wound site has been explored as treatment options for wound healing. Becaplerim, a gel containing recombinant platelet-derived growth factor (PDGF), was the first example of this approach and was approved by the FDA in 1997. Other available treatments that utilize growth factors and chemokines are Platelet-rich plasma (PRP) and amniotic membrane allografts (reviewed in [6]). PRP contains the plasma fraction with high concentrations of platelets and thereby increases the amount of growth factors such as TGF-β, PDGF, and epidermal growth factor (EGF) in the wound following release from platelets [43,44]. Amniotic membranes allografts have in multiple studies been shown to have a clinical effect on non-healing wounds, and there are some commercially available options (EpiFix, MiMedx Group, and the Grafix, Osiris Therapeutics) [45]. The amniotic membrane contains a large amount of growth factors, chemokines, and cytokines, including CXCL12 [46]. To utilize the inherent healing properties of wound macrophages, we recently developed an immunotherapeutic approach to kick-start wound healing. Thus, accelerated wound healing of full-thickness wounds was detected in mice following treatment with murine CXCL12-producing *L. reuteri* R2LC. The accelerated wound healing was most obvious in the days following wound induction, as the area of treated wounds was reduced by 50% compared to untreated or wild-type *L. reuteri* treated wounds [8]. In addition, the wounds treated with murine CXCL12-producing *L. reuteri* R2LC healed on average almost 2 days faster. As a major treatment effect in the mice was seen in the early phase of wound healing, contraction of the wound could be contributing to the observed strong effects, reinforcing the need to test the same therapy in minipigs. Indeed, in the current study in minipigs, the initial reduction of wound size is not seen, even though treatment with ILP100 (freeze-dried formulation of human CXCL12-producing *L. reuteri* R2LC) still reduces the number of days to complete re-epithelialization. Here, the effect on shortened time to wound healing was demonstrated in two separate cohorts, as Cohort A reports 3 days’ faster complete re-epithelialization using planimetry, while Cohort B demonstrates a reduced number of days to 50% and 75% re-epithelialization measured from 2D photographs. In fact, the human variant CXCL12 has previously been shown to have a biological effect on wound healing in Yorkshire pigs where healing was accelerated in full-thickness incision wounds treated with scaffolds soaked in human CXCL12 protein or plasmid DNA coding for CXCL12 [47].

In the current studies, both the presence of granulation tissue and the proportion of the wound cavity filled with granulation tissue were scored, with a similar scoring system to BWAT. In addition, the area of the wound covered with granulation tissue was measured by planimetry. We found that wounds treated with ILP100 demonstrated an accelerated formation of granulation tissue, shown by increased granulation scoring on day 5 and day 7 (Cohort A) and day 7 and day 9 (Cohort B), as well as increased area covered with granulation tissue on day 5 (Cohort A). Both studies revealed a reduced number of days to reach a granulation score of 4, indicating that the whole cavity is filled with granulation tissue more quickly. During wound healing, TGF-β is one of the major growth factors stimulating the formation of granulation tissue, and is released by wound macrophages and following degradation of extracellular matrix (ECM) [48,49]. Even though macroscopic evaluation of the wound provides important information on the healing process, and there are several validated tools for this as mentioned above, these evaluations are not traceable. Using the Cherry Imaging system, the healing of the cavity may be evaluated objectively by measurement of the depth and volume as well as assessment of the scar height and volume. In accordance with the granulation scoring, the 3D scans revealed reduced depth of wounds at day 9 following treatment with ILP100 when compared to the placebo. 

One limitation of the current experimental design is that the two cohorts were studied 2 years apart, which might have resulted in a slight shift in the grading criteria of the granulation tissue. Further, Cohort A included only male pigs whereas Cohort B was conducted in only females. However, all animals in the respective cohort were included in the study within a week and housed in the same stables, limiting the environmental differences and allowing for intra-cohort comparisons. Despite some observed differences in wound healing between the two cohorts following no treatment or treatment with placebo or wild-type bacteria, similar results were observed for treatment efficacy of ILP100. Thus, ILP100-treated wounds in two separate cohorts demonstrated the accelerated formation of granulation tissue and re-epithelialization, signifying the translational potential of our previous observations in mice.

## 5. Conclusions

We found that topical treatment with the new drug candidate ILP100 to full-thickness wounds in minipigs accelerates healing and is well tolerated. The current study also reveals the need for standardized methods to assess wound healing since differences between methods can be substantial. In addition to educating evaluators to use the same criteria for wound assessment, careful consideration should be taken when choosing methods, including the need for high accuracy, mode of action of the drug candidate, user-friendliness, traceability, and costs, as well as the risk of infection or other disturbances to the wound healing process.

## 6. Patents

ILP100 for the treatment of wounds is protected by patents in multiple countries. 

## Data Availability

The data presented in this study are available on request from the corresponding author.

## Appendix A

**Table A1:** Organs and tissues examined by histopathology in cohort A and B. * Indicate unilateral sampling and ** bilateral sampling.

Organs and Tissue for Histopathology	
Abnormalities (gross lesions)	Muscle, skeletal *
Artery, aorta (thoracic and aortic arch)	Nerve, optic **
Bone marrow, sternum	Nerve, sciatic *
Bone, femur (medial condyl) *	Pancreas
Brain	Skin, untreated
Esophagus	Small intestine, duodenum
Eye (with lens) **	Small intestine, jejunum
Gallbladder	Small intestine, ileum
Gland, adrenal **	Spinal cord, thoracic
Gland, mammary ** (Cohort A only *)	Spinal cord, lumbar
Gland, parathyroid (when possible) **	Spleen
Gland, pituitary	Stomach
Gland, salivary, parotid *	Thymus
Gland, salivary, sublingual *	Tongue
Gland, salivary, submandibular *	Ureter **
Gland, thyroid	Urinary bladder
Heart	Wounds
Joint, femorotibial *	
Kidney **	**Cohort A**
Large intestine, cecum	Gland, prostate
Large intestine, colon	Testis
Large intestine, rectum	
Larynx	**Cohort B**
Liver (all main lobes)	Lymph node, axillary
Lung (cranial and caudal lobes, both sides)	Ovary **
Lymph node, mandibular *	Uterus (horn **, cervix and oviduct **)
Lymph node, mesenteric	Vagina

**Table A2:** Average score (0–4) for wound edge inflammation and inflammation in the surrounding skin for untreated wounds, wounds treated with wild type *L. reuteri* R2LC or ILP100 in Cohort A and wounds treated with placebo or ILP100 wounds in Cohort B. For time points not included in the table all wounds in all groups were scored as 0.

Wound Edge Inflammation (Mean ± SEM)					
**Cohort A**	**Day 3**	**Day 5**	**Day 7**	**Day 9**	***n*;*N***
Untreated	0.17 (± 0.09)	0.00 (± 0.00)	0.17 (± 0.09)	0.00 (± 0.00)	3;18
Wild type R2LC	0.33 (± 0.14)	0.00 (± 0.00)	0.00 (± 0.00)	0.00 (± 0.00)	3;12
ILP100	0.40 (± 0.09)	0.17 (± 0.69)	0.13 (± 0.8)	0.14 (± 0.07)	6;30
**Cohort B**	**Day 3**	**-**	**Day 7**	**Day 9**	***n*;*N***
Placebo	0.11 (± 0.08)	-	0.00 (± 0.00)	0.00 (± 0.00)	3;18
ILP100	0.19 (± 0.07)	-	0.06 (± 0.04)	0.00 (± 0.00)	6;36
**Surrounding Skin Inflammation (Mean ± SEM)**					
**Cohort A**	**Day 3**	**Day 15**	**Day 21**	**Day 29**	***n*;*N***
Untreated	0.11 (± 0.08)	0.00 (± 0.00)	0.00 (± 0.00)	0.00 (± 0.00)	3;18
Wild type R2LC	0.25 (± 0.45)	0.00 (± 0.00)	0.25 (± 0.45)	0.58 (± 0.29)	3;12
ILP100	0.03 (± 0.03)	0.00 (± 0.00)	0.13 (± 0.06)	0.57 (± 0.18) *	6;30
**Cohort B**	**Day 3**	**Day 15**	**Day 21**	**Day 28**	***n*;*N***
Placebo	0.00 (± 0.00)	0.22 (± 0.10)	0.17 (± 0.09)	0.11 (± 0.08)	3;18
ILP100	0.00 (± 0.00)	0.00 (± 0.00) *	0.00 (± 0.00) *	0.00 (± 0.00)	6;36

**Table A3:** Average score (0–4) for hemorrhage for untreated wounds, wounds treated with wild type *L. reuteri* R2LC or ILP100 in Cohort A and wounds treated with placebo or ILP100 wounds in Cohort B. For time points not included in the table, all wounds in all groups were scored as 0. * *p* < 0.05.

Hemorrhage (Mean ± SEM)								
**Cohort A**	**Day 2**	**Day 3**	**Day 5**	**Day 7**	**Day 9**	**Day 11**	**Day 13**	***n*;*N***
Untreated	0.50 (± 0.62)	0.22 (± 0.10)	0.33 (± 0.11)	0.28 (± 0.14)	0.89 (± 0.08)	0.11 (± 0.08)	0.03 (± 0.03)	3;18
Wild type R2LC	0.75 (± 0.22)	0.08 (± 0.08)	0.33 (± 0.14)	1.42 (± 0.15) *	0.83 (± 0.17)	0.00 (± 0.00)	0.00 (± 0.00)	3;12
ILP100	0.17 (± 0.07)	0.10 (± 0.06)	0.23 (± 0.08)	1.17 (± 0.11) *	0.30 (± 0.09) *	0.00 (± 0.00)	0.00 (± 0.00)	6;30
**Cohort B**	**-**	**Day 3**	**-**	**Day 7**	**Day 9**	**Day 11**	**Day 13**	***n*;*N***
Placebo	-	1.17 (± 0.12)	-	0.39 (± 0.12)	0.56 (± 0.12)	0.61 (± 0.12)	0.11 (± 0.08)	3;18
ILP100	-	0.47 (± 0.13) *	-	0.94 (± 0.09) *	0.47 (± 0.08)	0.22 (± 0.07) *	0.06 (± 0.04)	6;36

**Table A4:** Average score (0–4) for exudate for untreated wounds, wounds treated with wild type *L. reuteri* R2LC or ILP100 in Cohort A and wounds treated with placebo or ILP100 wounds in Cohort B. For time points not included in the table, all wounds in all groups were scored as 0. * *p* < 0.05.

Exudate (Mean ± SEM)									
**Cohort A**	**Day 2**	**Day 3**	**Day 5**	**Day 7**	**Day 9**	**Day 11**	**Day 13**	**Day 19**	***n*;*N***
Untreated	1.28 (± 0.11)	1.39 (± 0.16)	1.72 (± 0.11)	0.61 (± 0.12)	0.39 (± 0.14)	0.17 (± 0.09)	0.00 (± 0.00)	0.00 (± 0.00)	3;18
Wild type R2LC	0.83 (± 0.11) *	0.67 (± 0.14) *	0.92 (± 0.15) *	0.17 (± 0.11)	0.25 (± 0.13)	0.08 (± 0.08)	0.00 (± 0.00)	0.08 (± 0.08)	3;12
ILP100	0.63 (± 0.09) *	0.67 (± 0.10) *	0.80 (± 0.12) *	0.57 (± 0.11)	0.50 (± 0.10)	0.03 (± 0.03)	0.07 (± 0.05)	0.00 (± 0.00)	6;30
**Cohort B**	**-**	**Day 3**	**-**	**Day 7**	**Day 9**	**Day 11**	**Day 13**	**-**	***n*;*N***
Placebo	-	1.56 (± 0.12)	-	1.17 (± 0.17)	0.56 (± 0.12)	0.00 (± 0.00)	0.00 (± 0.00)	-	3;18
ILP100	-	1.50 (± 0.08)	-	0.75 (± 0.11)	0.47 (± 0.08)	0.17 (± 0.06)	0.06 (± 0.04)	-	6;36

**Table A5:** Average score (0–4) for hypergranulation for untreated wounds, wounds treated with wild type *L. reuteri* R2LC or ILP100 in Cohort A and wounds treated with placebo or ILP100 wounds in Cohort B. For time points not included in the table, all wounds in all groups were scored as 0. * *p* < 0.05.

Hypergranulation (Mean ± SEM)				
**Cohort A**	**Day 7**	**Day 9**	**Day 11**	***n*;*N***
Untreated	0.00 (± 0.00)	0.17 (± 0.09)	0.06 (± 0.06)	3;18
Wild type R2LC	0.25 (± 0.13)	0.17 (± 0.11) *	0.00 (± 0.00)	3;12
ILP100	0.00 (± 0.00)	0.00 (± 0.00)	0.00 (± 0.00)	6;30
**Cohort B**	**Day 7**	**Day 9**	**Day 11**	***n*;*N***
Placebo	0.00 (±0.00)	0.00 (±0.00)	0.33 (±0.11)	3;18
ILP100	0.03 (±0.03)	0.08 (±0.05)	0.22 (±0.07)	6;36
